# Evaluation and cultivation method of high-tech value patents for mechanical products

**DOI:** 10.1371/journal.pone.0298144

**Published:** 2024-03-04

**Authors:** Chuan He, Fan Shi, Runhua Tan

**Affiliations:** 1 College of Mechanical and Electrical Engineering, China Jiliang University, Hangzhou, China; 2 National Engineering Research Center for Technological Innovation Method and Tool, Tianjin, China; 3 College of Civil Engineering and Architecture, Zhejiang University of Water Resources and Electric Power, Hangzhou, China; East China Normal University, CHINA

## Abstract

Evaluation of high value patents is essential for the enterprise’s technical layout and innovative product design. The existing research on the patent value needs the support of a large number of professional statistical information and is difficult to directly reflect the technical value. Since technological innovation is the fundamental means to enhance the sustainable competitiveness of enterprises. Therefore, a high-tech value patent evaluation and cultivation method for engineering designers need to be proposed. Firstly, the patent samples based on design methodology are retrieved and the indicators for evaluating technical value are summarized and the rationality of the evaluation indicators is verifier through empirical study based on improved evidence theory. Secondly, based on principal component analysis and factor analysis, a high-tech value patent evaluation and cultivation method is proposed. Finally, the proposed method is applied to identify the high-tech value patents in the cutting machine industry, and structural improvement is made based on this patent to demonstrate the cultivation process of high-tech value patents. The proposed method provides a clear guiding direction for the cultivation of high novelty patents and enterprise innovative product design. The method can effectively assist the product R&D activities of engineering designers and enhance the sustainable competitiveness of enterprises from a technological perspective.

## 1 Introduction

Innovation is the primary driving force for development, and patents are the main representative of technological innovation. The creation and application of intellectual properties are key components of an industry to keep its competitive position in the market. Patent information is an important form of the intellectual properties [1]. Using the patent information can shorten 60% of the R&D time and save 40% of the research funds for industries [2]. According to a survey of the world intellectual property organization, 90% - 95% of inventions can be found in patent documents, but 80% of them are not recorded in other documents. Normally, inventions appear in patent documents 1–2 years earlier than in other media. Therefore, the patent information analysis becomes more and more important for the technological innovation [3]. Patent information has been widely used in the development and evaluation of industrial products [4] and technology life cycle prediction [5]. The large number of patents with low creativity and legally invalid that abound in the industry will cause enterprise managers to misjudge the direction of product R&D. The structure improved based on low novelty patents will also not be competitive. Therefore, the screening of high technical value patents is of great practical importance for product R&D.

The evaluation of high-value patents has a benchmarking and leading effect on the R&D direction of the industry. High value patents are an important indication of the birth of innovative technologies. Scheme evaluation in conceptual design stage is an effective way for successful implementation [6]. The evaluation of high value patents is the main content in the conceptual design stage [7]. The high value patents account for only about 10% of the total number of patents, but their total value accounts for more than 80% [8]. American IP Consulting and the National Science Foundation have jointly developed the world’s first patent evaluation system, which enables the evaluation of the overall strength of a company’s or country’s and region’s intellectual property rights by evaluating the value of an intangible assets [9]. The evaluation system contains seven indicators: number of patents, average number of patent citations, current impact index, technological strength, technological life cycle, scientific relevance, and scientific strength.

The existing research on patent value evaluation methods mainly focuses on three dimensions of technology, law and market [10]. Although there is no unified definition of high value patents in academic circles, the evaluation method based on statistical information in three dimensions is the most widely accepted view. The view holds that high value patents should have significant technological breakthroughs and produce unexpected technical effects. From the legal perspective, the patent should be able to withstand the test of legal review, legal proceedings and other trial procedures. From the market perspective, high value patents can bring high benefit returns to their affiliated enterprises [11]. You [12] studied the patent value evaluation model from a technical perspective in combination with the content of the patent text, and the automated evaluation process using deep learning and NLP techniques can effectively reduce the workload of manual annotation. Gong [13] described the innovation value chain in terms of universities operating patents, and the study concluded that university patent commercialization activities can effectively feed back the innovation value of patents. Chen [14] used artificial neural networks to explore the influences of patent counts, defendant counts of patent litigation, and patent share on market value in the American semiconductor industry. Su [15] established a relative network connection based on the litigation patent to locate the high value patent through the key chain.

In addition to scholars’ research on patent value from the perspective of law, business and technology, the government has also screened high value patents from its own perspective. In order to encourage enterprises to realize independent R&D innovation of products and enhance the comprehensive competitiveness of local enterprises. The science and technology departments of governments at all levels select a number of innovative enterprises with high value patents by setting up science and technology progress awards. However, the screening of high-value patents from the government’s perspective is more focused on the consideration of economic benefits in terms of revenue generation. The event of transformation of scientific and technological achievements or the manufacturing behavior of products based on patents are important basis for the government to select high value patents. These awarded patents comprehensively reflect the technological layout and market operation of enterprises. For example, Huang [16] investigated more than 70,000 invention patents, used the yield of patent commercialization as a measure of the level of patent innovation, and proposed strategies to promote the yield of patents from the perspective of R&D resource allocation.

For product development-oriented enterprises, technological innovation is an effective means to maintain their sustained competitiveness[17]. Based on the evaluated high-tech value patents, it helps to design more innovative product structures. However, the existing methods for analyzing patent value based on statistical data can hardly reflect the level of technological innovation of patents directly. The deficiency of existing research leads to the blindness of enterprises in patent retrieval, and the improvement objects retrieved lack substantive innovation, which seriously hinders the further development of enterprises. The contribution of this article is to construct a systematic method for evaluating the technical value of patent, which is used by designers in the engineering field for product innovation activities. Propose a patent technical value evaluation method for engineering designers, which can help improve product R&D efficiency and enhance the innovation of structural design. Therefore, the aim of this study is to propose a new method for evaluating and cultivating high-tech value patents, which has important practical value for product development.

The innovation design process of mechanical products has developed from intuition and experience to multidisciplinary intersection and systematic design, emphasizing more and more the scientific, systematic and innovative nature of the design process. The traditional innovation methods represented by brainstorming and trial-and-error methods are inefficient due to the lack of systematic solutions to problems and the scattered design process. Therefore, it is necessary for technical personnel to master systematic innovative design methods and processes, so as to design mechanical products that meet user needs with highly innovative. Design methodology is an important discipline used to guide product innovation design. It contains numerous design theories: substance-field model, technique conflict, physical conflict, avoiding design, the law of technological evolution, the scientific effect, the ARIZ algorithm, and so on. Searching with multiple tools in design methodology as keywords can obtain patents on product structure innovation. Therefore, a lot of knowledge from design methodology will be applied to retrieve high-tech value patents and serve as samples for analysis.

The rest of this paper is organized as follows: Section 2 reviews a variety of methods for evaluating patent value. Section 3 constructs a patent technique value evaluation method. Section 4 illustrates the validity of the proposed evaluation method through empirical analysis. Section 5 discusses the application process of the proposed evaluation method in the product innovation design. Section 6 shows the contributions and shortcomings of the method and suggests future work.

## 2 Related research

Taking high-value patents as the starting point of enterprise technology R&D can effectively shorten the product development cycle and enhance the technological competitiveness of enterprises. Therefore, the evaluation of high technical value patents is particularly important.

### 2.1 Patent value evaluation methods

(1) Patent indicator method

Numerous scholars study on the indicators affecting patent value: patent life cycle, patent protection scope, patent inventiveness, patent R&D investment, and patent owner [18]. These studies have explored the factors affecting the value of patents from the perspectives of single and multiple indicators. Dechezleprêtre [19] improved the evaluation indicator of the number of patent families and added the indicator of the number of patent applications of priority countries within the patent families. Berger [20] found that the number and frequency of high value patents modifying claims in the examination stage is higher than that of ordinary patents through statistics of EPO patent application process data, which proves that the number of claims is positively related to the value of patents.

Considering that a single indicator is difficult to describe the patent value comprehensively, the patent value evaluation model containing multiple indicators has received much attention. The Manual of Patent Value Analysis Index System Operation, jointly published by the State Intellectual Property Office of China and the China Technology Exchange, has become the main reference for many scholars to evaluate the value of patents. It contains a total of 18 evaluation indicators, and the degree of contribution of each indicator to patent value (0–100) through expert scoring [21]. Park [22] established a two-dimensional patent value evaluation system that includes technical intrinsic elements and application elements. Wan [23] obtained three indicators that can express the value of patents: the number of claims, the number of citations, and the number of inventors through the statistical analysis of a large number of patents in China and the United States. Liu [24] used a morphological matrix method to assess patent values in combination with the patent novelty and compatibility. Hirschey [25] used scientific value indicators such as technology life cycle, citation rate and non-patent literature to assess patent value. Ernst [9] combined four indicators of authorization rate, efficiency, cited rate and the United States share to characterize patent values. Chang [26] divided the indicator affecting patent value into four categories of quality, technology, value and management to construct a patent value indicator system consisting of four secondary indicators and twenty-two tertiary indicators. Wu [27] proposed two indicators, patent family depth and earn plan ratio, both of which are positively correlated with patent value.

Establishing a comprehensive measurement patent value network based on evaluation indicators is also an important method. For example, Kim [28] calculated the centrality value of patents to analyze the network of technical components and select patents that record key technologies. Hu [29] established a patent centripetal reference network among patent value indicators, and applied Monte Carlo simulation to simulate the mapping relationship between these indicators and patent values. Huang [30] explored the factors influencing patent value in the LED industry in terms of extra-degree centrality, independent centrality, proximity centrality and network location using a logistic regression model, and the empirical results showed that extra-degree centrality and endo-degree centrality had a significant positive effect on patent value, while network location had a significant negative effect on patent value. In addition, a probabilistic graph-based patent valuation model was proposed for automatic patent value assessment. This model uses a heterogeneous association network as an evaluation scenario and reasoning with the valuation model to derive the patent value distribution [31].

(2) Cooperation of inventors

The relationship between inventors’ cooperation and patent value has also attracted scholars’ attention. For example, to address the issue of the relationship between patent co-ownership of SMEs and the company’s market value, Lv [32] found that joint patenting intensity was negatively correlated with the market value of SMEs, and government subsidies strengthen the above negative correlation. Lo [33] analyzed the global patent collaboration R&D of nanotechnology and investigated the influence of global R&D collaboration on patent value. He finally concluded that collaboration among star assigners, global partnership, and patent centrality, which have a positive relationship on patent value.

The technology transfer behavior of innovation achievements and the exchanging of patents activities among inventors are considered to describe the market value of patents [34]. Eom [35] proposed a model to predict the market value of patents by collecting patent transaction events and using an ensemble learning methodology. Breitzman [36] found that high-value patents all acquired more than eight co-inventors by calculating the size of inventor teams for the frequency cited over a five-year period. Du [37] pioneered the use of patent inventors’ reputation as a new indicator of patent value, and proposed a method to push high-value patents to target buyers by analyzing the patent citation network of potential buyers.

(3) Patent citation method

Patent citation is a method to evaluate the patents value by constructing the citation relationship between patents. Narin [38] conducted research on the patent metrology based on bibliometrics, and found that patent citation can represent the importance of technologies. Atallah [39] analyzed the relationship between the number of citations and value of patents, and considered the issue of patent citations under the influence of time. They developed a citation indicator for more than 2 million patents in the United States to verify its effectiveness. Lee [40] determined that there was a positive relationship between the patent citation and patent value according to the patent application of Korea Institute of Science and Technology. You [41] built a citation network among patents to assess patent values by building topology. Bakker [42] found that there was a logarithmic relationship between the number of patent citations and patent values through the analysis of a forward citation indicator of patents. Chang [26] employed the panel threshold regression model to examine the influence of the structure of the patent citation network on the patent value, and concluded that there is no linear relationship between the patent ranking and the patent value constructed by the citation network. To characterize the inherent attractiveness of the patents to be cited, a fitness parameter was constructed. The fitness parameter constructed by the patent family specifies the changing law of the citation network over time [43]. Leila [44] found a positive correlation between patent forward citation data and patent value by investigating the share of USPTO, EPO and JPO patents in the patent family. Bakker [45] found that the relationship between patent citation and patent value is log-linear through empirical analysis. In order to more accurately express the knowledge flow information among patents, Yang [46] combined four citation networks (direct citation, indirect citation, coupling and co-citation networks), and proposed a comprehensive patent citation network for evaluating patent value. Chang [47] used a panel threshold regression model to test the threshold effect of the patent H-index on the relationship between patent citations and market value in the pharmaceutical industry. Due to the differences in patent citation by patent applicants and examiners, Park [48] proposed a new perspective to analyze patent citation and concluded that the quality of patents cited by applicants is higher than that of patents cited by examiners.

In summary, existing patent value evaluation methods have their own pros and cons. In the existing patent value evaluation system, a large number of evaluation indicators such as the degree of technological innovation and marketability are difficult to calculate or quantify, which poses a challenge to the quantitative evaluation of patent value. The patent value evaluation method based on citation relationship is applicable to patents filed in USPTO and EPO. However, similar to KIPO and SIPO, many patent statistics are not published, including the number of patent citations, legal proceedings, market share, patent enforcement rates of competing companies and related interests, which limits the scope of application of existing patent value evaluation methods. Indicators such as number of claims, number of inventors and cooperation of inventors are applicable to patents filed at any patent office and have the advantage of being widely applicable. While these indicators can describe patent value from a general statistical perspective, it is difficult to ensure that the screened patents carry highly innovative technical features.

Although the advantages of the existing methods are obvious and are suitable for the rapid processing of large volumes of patent data. However, it is difficult to directly identify patents that contain highly innovative technical features. For product R&D-oriented enterprises, structural improvement based on the technical features of a single high-tech value patent is a fast and efficient way to achieve a technological breakthrough. The widely accepted indicators for evaluating the technical value of patents are number of inventors, number of citations, number of classification codes, be cited numbers, number of claims [49]. However, these technical indicators belong to the category of statistical sense and do not belong to the technical meaning in the engineering field. Therefore, proposing a patent technical value evaluation method for engineering designers requires further research.

### 2.2 Patent indicator weights

Among various patent value evaluation methods, the patent indicator method is widely used by scholars. Moreover, compared with the single-indicator evaluation method, the patent value evaluation system that contains multiple indicators can calculate the patent value more accurately. The corresponding multiplication of indicators by their respective weights results in the patent value evaluation formula. The widely used method of determining indicator weights is obtained through the judgment of domain experts in scoring. Since there is an unavoidable subjective factor in the expert’s existence, the multi-attribute decision method is introduced into the study of determining indicator weights to compensate for the limitations caused by the exact numbers.

AHP is a convenient multi-attribute decision making method, which is useful to enhance the objectivity of indicator weight assignment. The method has been widely used in various fields such as environmental science, industrial decision making and health care. Sujian [50] proposed an integrated fuzzy AHP-Topsis method for the calculation of green scores in the hospitality industry containing 26 indicators to enhance the environmental capability of the hospitality industry. Billur [51] proposed an integrated AHP from the perspectives of legal, economic, physical factors and locational model that can consider multiple criteria simultaneously to determine the best investment option for real estate. Abdel [52] used the AHP-Topsis method to study the risks involved in the production process of the Indian textile industry and found that the top three indicators of importance were lighting, ventilation, and noise. Salehi [53] used the Entropy-Topsis method to assess the risk management of the fossil industry and the two most important indicators were obtained as management capacity and human factors.

Although, there is inevitably personal subjectivity in determining indicator weights through expert scoring. However, it must also be recognized that the product development process cannot be separated from the subjective creativity of experts. In the process of patent sample evaluation, relying on expert scoring is an unavoidable step. For example, Liu [54] invited one expert in the field of additive manufacturing to build the technological change value metrics for the three design cases. Wang [55] also invited one expert to score the changes in the four product subsystems in order to evaluate the radial innovation product. The operation of relying on a single expert for evaluation has been accepted, but the method of increasing the number of experts to enhance the objectivity of the results still needs to be studied.

It has become a trend to apply multi-attribute decision models to the indicator weight determination process, which helps to improve the accuracy. However, the fuzzification of data still makes it difficult to address the subjectivity that necessarily exists for a single decision maker. Therefore, more than two decision makers should be invited and scored simultaneously for the same event to compensate for the bias of a single individual. However, the existing operations of fusing the scores of multiple experts mostly use the mean calculation method, which lacks a scientific mathematical calculation theory. Therefore, the research from the perspective of fusing the scoring results of multiple experts to improve the objectivity of indicator weights still needs further improvement.

Therefore, the efforts will be made in both the objectivity of scoring and the evaluation of the technical value of patent. In order to screen out the patents with high innovative technical features, this paper proposes a high-tech value patent (HTVP) evaluation method for engineering designers. The method can help designers locate the benchmark technologies in the industry, and by analyzing the technical features of the screened HTVPs, and then combining them with customer needs in the market for structural improvement designs, more innovative patented products can be obtained. The proposed method points out a reference idea for the cultivation of HTVP, which has strong practical significance for R&D enterprises.

## 3 Patent technical value evaluation

**Fig 1:**
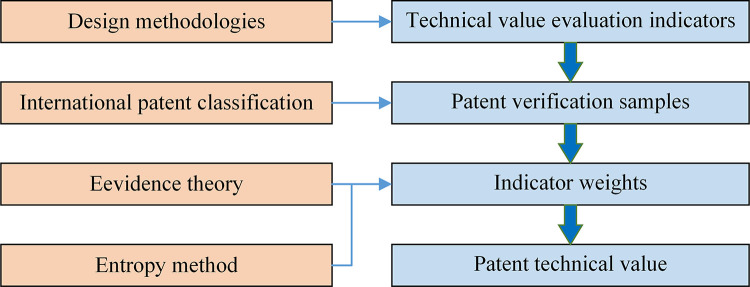
The process of the patent technical value evaluation.

In order to directly measure the level of patent technological innovation and to provide direction for the cultivation of HTVP, especially to meet the increasing demand for patent applications and intellectual property protection in Chinese industries. Indicators for evaluating technological innovations are summarized, and patents for mechanical products filed at the State Intellectual Property Office are collected as verification samples. The patent verification samples (PVS) will be applied to verify the rationality of the proposed technical evaluation indicators (TEI). Patent technical value evaluation method will eventually be constructed. The construction process of the patent technical value evaluation method is shown in Fig 1.

### 3.1 Technical value evaluation indicators

This section will creatively propose a technical evaluation indicator acquisition method for engineers from the perspective of high technology value patents. Analyzing the technical characteristics of high-tech value patents will improve the R&D efficiency of product innovation design. Therefore, this section will collect high technical value patents and summarize the technical characteristics of these patents, and then transform into indicators for evaluating the patent technical value.

The establishment of scientific and reasonable evaluation indicators is an important part of evaluating the patent technical value. The patent technical value evaluation indicator system for mechanical products should not only meet the requirements of comprehensiveness and scientificity, but also follow the relevant theoretical connotation and practical needs, and ensure the applicability of the evaluation indicator system in practical work.

Since academics have not yet formed a unified definition of high technology value patents, a new method of patent collection will be proposed. Although the award-winning patents reflect a comprehensive value, it is difficult to directly reflect the technical value from the perspective of structural features. Therefore, the method for evaluating patent value focuses on the quality from a technical perspective need to be proposed. Articles with design methodologies in the field of engineering that deal with innovative tools are searched. Design methodology is an important discipline used to guide product innovation design. It contains numerous design theories [56]: substance-field model, technique conflict, physical conflict, avoiding design, the law of technological evolution, the scientific effect, the ARIZ algorithm, and so on. Many scholars have improved the theories of design methodology in order to redesign the product structure and published them in papers. Through the continuous improvement of design methodology, more innovative product structures can be further designed. The patents with high structural innovativeness recorded in the papers are extracted, which reflect high technical value. In order to eliminate regional differences in patent filings, only the patents filed at the SIPO will be retained.

A patent based on an improved structure will have a higher technical value. Therefore, Patents documented in published articles are focused on and technical features are extracted based on these patents. The search terms for the relevant subject articles are as follows: TRIZ, Patent design around, Function trimming, Axiomatic design, Anticipatory failure determination, Quality function deployment, Technological evolution, Technical conflict. The search terms are restricted to the article titles, and a total of 623 articles are retrieved. Patents marked with application numbers and applied for in the SIPO are extracted from these articles. There are many articles that show improvements in product structure, however, few clearly show the patent application number in those articles. Finally, a total of 29 patents belonging to the field of mechanical engineering are collected. Although these patents focus on different innovations, through comparative research, it is found that these patents have common characteristics in high technical values and similarities in innovation mechanisms to represent the common characteristics of high value patents. The collected representative patents are listed in Table 1.

**Table 1:** Collected representative patents.

NO.	Application number	NO.	Application number	NO.	Application number	NO.	Application number
1	CN200420018862.9	9	CN201010134223.9	17	CN201520113620.6	25	CN01812693.6
2	CN03239424.1	10	CN200710093247.2	18	CN201020663432.8	26	CN201420320360.5
3	CN00226535.4	11	CN200510057124.4	19	CN201420515518.4	27	CN200720022017.2
4	CN02275538.1	12	CN201320194177.0	20	CN200520031671.0	28	CN201320586988.5
5	CN201210529974.X	13	CN200820179326.5	21	CN02815101.1	29	CN201110007817.8
6	CN200420063217.9	14	CN200810054176.X	22	CN99110940.6		
7	CN94211164.8	15	CN200820216662.2	23	CN00803249.1		
8	CN94211164.8	16	CN201410310309.0	24	CN01814244.3		

**Fig 2:**
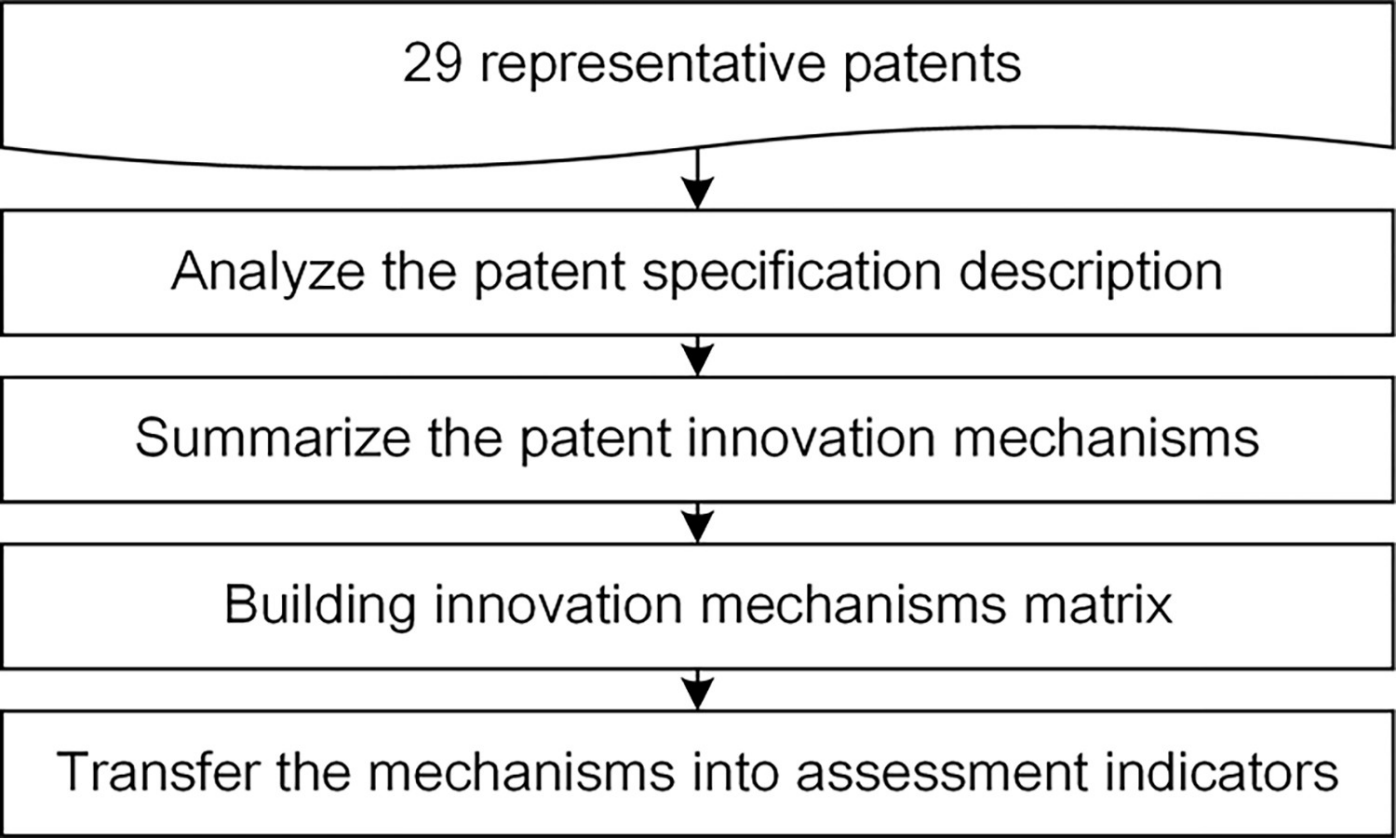
Construction of evaluation indicators.

The collected patent documents are read one by one and the respective technical features are summarized. The innovation mechanisms of these patents are summarized based on the analysis of technical features and translate into indicators that can describe technical innovation [57]. The process of construction of evaluation indicators is shown in Fig 2.

It should be noted that the construction process of the patent technical value evaluation indicators is an iterative process. This is because the number and content of the evaluation indicators cannot be predicted and known until the evaluation indicator data is finally obtained. An evaluation indicators construction matrix method is proposed, which can be seen in Table 2.

**Table 2:** Evaluation indicators construction matrix.

Patent number	Technical indicator number																	
	A	B	C	D	E	F	G	H	I	J	K	L	M	N	O	P	Q	R
1	1	0	0	1	0	0	1	1	0	1	1	0	1	0	0	0	1	0
2	0	0	0	1	0	0	1	0	0	0	1	0	1	0	0	0	0	0
3	1	0	1	0	0	0	0	0	1	0	0	0	0	0	0	0	0	0
4	0	0	1	0	0	0	0	0	0	0	1	0	0	0	0	1	0	0
5	0	0	1	0	0	0	0	1	0	0	0	0	1	0	0	0	0	0
6	0	0	1	0	0	0	0	0	0	0	1	0	1	1	0	1	0	1
7	1	0	1	1	0	0	0	0	0	0	1	0	0	0	0	0	1	0
8	0	0	1	0	0	0	0	1	0	1	0	0	0	0	0	0	0	0
9	0	0	0	0	1	0	0	0	0	0	0	0	0	0	1	0	1	0
10	1	0	0	0	1	0	0	0	0	0	1	0	0	0	1	0	0	0
11	1	1	0	0	1	0	1	0	0	0	0	1	0	0	1	0	0	0
12	0	1	0	0	0	0	0	0	0	0	0	0	0	1	1	0	0	0
13	0	0	1	0	0	1	0	0	0	1	0	0	0	0	0	0	0	0
14	0	1	0	0	0	0	0	0	1	0	0	1	0	0	0	0	0	0
15	0	0	1	0	0	0	1	0	1	0	1	0	0	0	0	0	0	0
16	0	1	0	0	0	1	0	0	1	0	0	0	0	0	0	0	0	1
17	0	1	0	0	0	1	0	0	0	0	0	0	0	0	0	1	0	0
18	0	1	0	0	0	1	0	0	0	0	1	0	0	0	0	0	0	0
19	0	1	0	0	0	1	0	0	0	0	0	0	0	0	0	0	1	0
20	0	0	0	1	0	0	0	0	0	1	0	1	0	0	0	0	0	0
21	0	1	0	0	0	0	0	0	1	0	1	0	0	0	0	1	0	0
22	0	0	0	0	0	0	0	0	1	0	0	0	1	0	0	1	0	1
23	1	0	1	0	1	0	1	0	0	0	0	0	0	0	0	1	0	0
24	1	0	0	0	0	1	1	0	0	0	0	0	0	1	0	0	0	1
25	1	1	0	0	1	0	1	1	0	1	0	0	0	0	0	0	0	0
26	1	0	0	0	1	0	0	1	1	0	0	0	0	0	1	0	0	1
27	0	1	0	0	0	0	0	0	0	0	0	0	0	1	0	0	1	1
28	0	0	0	1	0	0	0	0	0	0	0	1	0	0	0	0	0	1
29	1	0	0	0	0	0	1	0	1	0	1	1	0	1	1	0	1	1

The vertical coordinates are the patent serial numbers and the horizontal coordinates are the serial numbers of the indicators to be constructed. “1” indicates an applied indicator for related patents, and “0” represents no related indicator. Analyze the patent document in turn and construct evaluation indicators, and when new indicators appear in subsequent patents, go back to confirm the existence of such indicators in previous patents to prevent omission. For example, a total of 8 evaluation indicators are collected by analyzing the first patent, and 4 evaluation indicators are obtained by analyzing the second patent, which all overlap with the data from the first patent. The analysis of the third patent yield a total of 3 evaluation indicators, one of which overlap with the previous one, and two of which are new items. At this point it is necessary to go back and re-analyze the first two patents to see if they have the two new indicators add to the third patent. The above analysis process is iterative until all 29 patents have been analyzed. The matrix approach shown in Table 2 ensures that the matching data between the patent and the evaluation indicators is not missed.

Finally, 18 technical value evaluation indicators are obtained. The evaluation indicators based on the extraction of representative patents are shown in Table 3.

**Table 3:** Patent technical value evaluation indicators.

NO.	Technical value evaluation indicators	NO.	Technical value evaluation indicators
A	Perfect design function	J	Conform to the principle of assembly process
B	Accord with human body and sensory	K	Improve stability
C	Improve mechanical properties	L	Adopt advanced technology and materials
D	Improve reliability	M	Adopt automation and adaptive principle
E	Meeting the baseline principle to ensure assembly accuracy	N	Feedback, adjustable for various states
F	Quality and optimal design	O	In line with the principle of human-machine division of labor
G	Conform to the principle of standardization and function distribution	P	Reasonable formulation of process route and procedure
H	In accordance with parts and structure design process	Q	Good external characteristics
I	Excellent man-machine relationship	R	optimization of structural layout

### 3.2 Patent verification samples

In order to verify the rationality of the above-mentioned eighteen patent technical value evaluation indicators, verification samples of invention patents are selected according to the classification principle of International Patent Classification (IPC) [58]. The patent technical value evaluation indicators are scored through analyze each patent verification samples (PVS) in detail.

**Fig 3:**
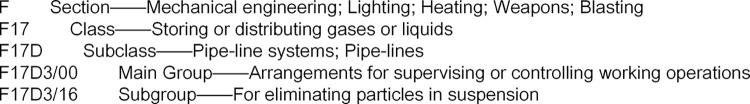
IPC classification level.

IPC was developed for the Strasbourg agreement on International Patent Classification signed in 1971. IPC can statistically classify a large number of patent documents as a universal patent classification method. The structure of the IPC classification number consists of section, class, subclass, main group, and subgroup. The lower the level, the more detail an area is divided. An IPC layering pattern with the pipeline observation system as an example is shown in Fig 3.

Selection rules of patent verification samples based on IPC are as follows:

1. Verification samples in Section F of mechanical engineering are selected. The patent verification samples should cover all sub-areas of Section F with innovative structural designs in mechanical engineering. Section F consists of seventeen classes, each of them contains a number of subclass ranging from one to fourteen. In order not to make the sample too large to process, it is determined that the lowest level of patent verification samples is the subclass. A sample of invention patents is randomly selected from each subclass.
2. In order to eliminate the difference of market values caused by application time, the application year of all verification samples is limited in 2018. The verification sample should be selected to maximize the elimination of possible adverse influences. The difference in the time of patent application will result in different market values of patents. In order to eliminate the influence caused by the market environment on the verification samples, the data of patents applied in the same year should be selected. Therefore, selecting the patent data applied in that year as the verification sample will have stronger objectivity.

According to the above rules, 96 invention patents are selected as verification samples arranged in alphabetical order according to the IPC classification number as shown in Table 4. The database applied for patent retrieval in this paper is Patsnap, whose web address is https://analytics.zhihuiya.com/search/input/simple.

**Table 4:** Patent verification samples.

NO.	Patent samples	NO.	Patent samples	NO.	Patent samples	NO.	Patent samples
1	CN109404046A	25	CN109356861A	49	CN109307165A	73	CN109028562A
2	CN109162762A	26	CN109268320A	50	CN109373217A	74	CN107465387A
3	CN109356674A	27	CN109372818A	51	CN109268770A	75	CN109386977A
4	CN109268087A	28	CN109340242A	52	CN109340693A	76	CN109373643A
5	CN109356682A	29	CN109340250A	53	CN108679504A	77	CN109282547A
6	CN109339902A	30	CN109372892A	54	CN108980799A	78	CN109373690A
7	CN109372617A	31	CN109372898A	55	CN109357249A	79	CN108562112A
8	CN109372632A	32	CN109372941A	56	CN108826271A	80	CN109341316A
9	CN109339955A	33	CN109611606A	57	CN109373309A	81	CN109341336A
10	CN109339965A	34	CN109322974A	58	CN109185872A	82	CN109323598A
11	CN109356743A	35	CN109340316A	59	CN109282281A	83	CN109387091A
12	CN109339967A	36	CN109253253A	60	CN109185899A	84	CN109373776A
13	CN109404160A	37	CN109373004A	61	CN108253408A	85	CN109297326A
14	CN109356746A	38	CN109373105A	62	CN109059033A	86	CN109084611A
15	CN109372669A	39	CN109372856A	63	CN109140484A	87	CN109387116A
16	CN109296488A	40	CN109340495A	64	CN109084323A	88	CN109141112A
17	CN109340017A	41	CN109295601A	65	CN109340817A	89	CN109282697A
18	CN109356775A	42	CN109323120A	66	CN109140500A	90	CN109186339A
19	CN109236559A	43	CN109237291A	67	CN109307286A	91	CN108917472A
20	CN109372696A	44	CN108361541A	68	CN109323287A	92	CN109341425A
21	CN109057926A	45	CN108488616A	69	CN109340860A	93	CN109297359A
22	CN108194295A	46	CN109386734A	70	CN109297053A	94	CN109341439A
23	CN109372713A	47	CN109323131A	71	CN109340882A	95	CN109163629A
24	CN109236654A	48	CN106856355A	72	CN109362215A	96	CN109141150A

### 3.3 Technical value evaluation based on the evidence theory and entropy method

Experts are invited to score patent verification samples based on the evaluation indicators to obtain the technical value score of each patent. The practice of relying on expert scoring to assess sample value has been recognized in the field of design methodology. For example, Liu [54] invited a single expert in the field of additive manufacturing to build the technological change value metrics for the three design cases. Wang [55] also invited a single expert to score the changes in the four product subsystems to evaluate the radial innovation products. However, the subject background of single expert may affect the scoring results. Therefore, two industry experts are invited to mark patents respectively. The invited experts need to have at least ten years of working experience in the field of mechanical design, and be familiar with the latest research and well-known technologies in the field. They should also can comment advantages and disadvantages of the new and existing designs [54]. Finally, the expert scoring results are fused with an improved evidence theory for further calculations. Compared to previous studies that invite only one expert, the proposed scoring method of the two experts will produce greater objectivity.

**Fig 4:**
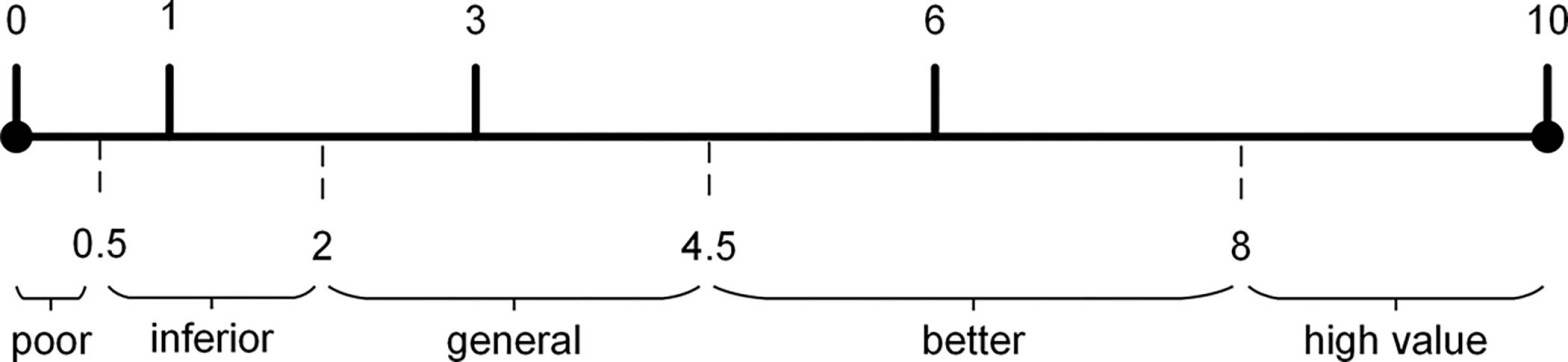
Patent value grades.

In order to enhance the theoretical basis for expert scoring, the technology genealogy tree method is introduced. Technology genealogy tree [59] is a tool to measure the degree of technology changes, which can describe technical differences from existing design schemes. The technology genealogy tree divides the technology difference degree into four levels of the physical principle, working principle, specific example and technical detail. Four levels are assigned scores of 10, 6, 3 and 1 respectively, which represent degrees of technology changes of the new design compared to the existing design. Each patent is scored according to the rule of technology genealogy tree from eighteen aspects of the evaluation indicator. For example, if a new design achieves new breakthroughs in the physical principle level compared to the existing design in order to achieve the same function, 10 points will be assigned. If the physical principle level does not change but the new working principle is used, 6 points will be given, and so on. Shang [60] divided the assessment of general things into five levels from poor to excellent through the network analysis. Based on the method of grading general things and the principle of genealogy tree, the patent value is divided into five grades: high, better, general, inferior, and poor as shown in Fig 4.

Based on scores of experts for patent P_*i*_ (*i* = 1, 2, 3 … 96) after compared evaluation indicators T_*j*_ (*j* = 1, 2, 3 … 18) with the existing design, the evidence theory is used to fuse scores of experts to eliminate the data dimension. The evidence theory, also known as D-S theory [61]. The method was first applied in the expert system to deal with uncertain information. The synthesis rule of evidence theory is as follows.

For ∀*A*⊆Θ, the combination of two mass functions *m*_1_, *m*_2_ on Θ is represented by Formula (1).

$$m_{1}⊕m_{2}(A)=\frac{1}{K}\sum_{B\cap C=A}m_{1}(B)•m_{2}(C)$$ (1)

where *K* is the normalization constant:

$$K=\sum_{B\cap C\neq \emptyset }m_{1}(B)•m_{2}(C)=1-\sum_{B\cap C=\emptyset }m_{1}(B)•m_{2}(C)$$ (2)

Two experts score all PVSs respectively, forming two scoring matrices *P*_96×18_ and *Q*_96×18_ in 96 rows and 18 columns. Since the evidence theory can only deal with the data fusion for an uncertain problem, the evidence theory needs to be improved. Based on the existing D-S theory research, a method suitable for processing two-dimensional matrix data fusion will be proposed. The improvement process of evidence theory is described below.

As patents need to be analyzed have different technical features, each subclass indicates different technical detail. Scoring results of different technical areas have different dimensions and orders in magnitude. In order to avoid the phenomenon of highlighting indicators weakening the lower indicators in the fusion process, it is necessary to normalize the original data to eliminate the data dimension. The normalization method is as follows.

$$s_{i}=\sum_{j=1}^{m}p_{ij}(j=1,2,3\ldots m)$$ (3)

$$p_{ij}^{'}=\frac{p_{ij}}{s_{i}}(i=1,2,3\ldots n;j=1,2,3\ldots m)$$ (4)

where *s*_*i*_ is sum of rows of matrix *p*_*ij*_. $p_{ij}^{'}$ is a normalization matrix.

After normalizing the scoring matrix *P*_96×18_ and *Q*_96×18_, matrix *R*_*n*×*m*_ is obtained using Formula (5) to calculate the scalar product of the scoring matrix.


$$r_{ij}=p_{ij}^{'}\times q_{ij}^{'}(i=1,2,3\ldots n;j=1,2,3\ldots m)$$ (5)

Fusion matrix $R_{n\times m}^{'}$ is obtained by multiplying matrix *R*_*n*×*m*_ and normalization constant *K* using Formulas (6) and (7).


$$r_{ij}^{'}=\frac{1}{k_{j}}(i=1,2,3\ldots n;j=1,2,3\ldots m)$$ (6)

$$k_{j}=\sum_{j=1}^{m}r_{ij}(j=1,2,3\ldots m)$$ (7)

**Fig 5:**
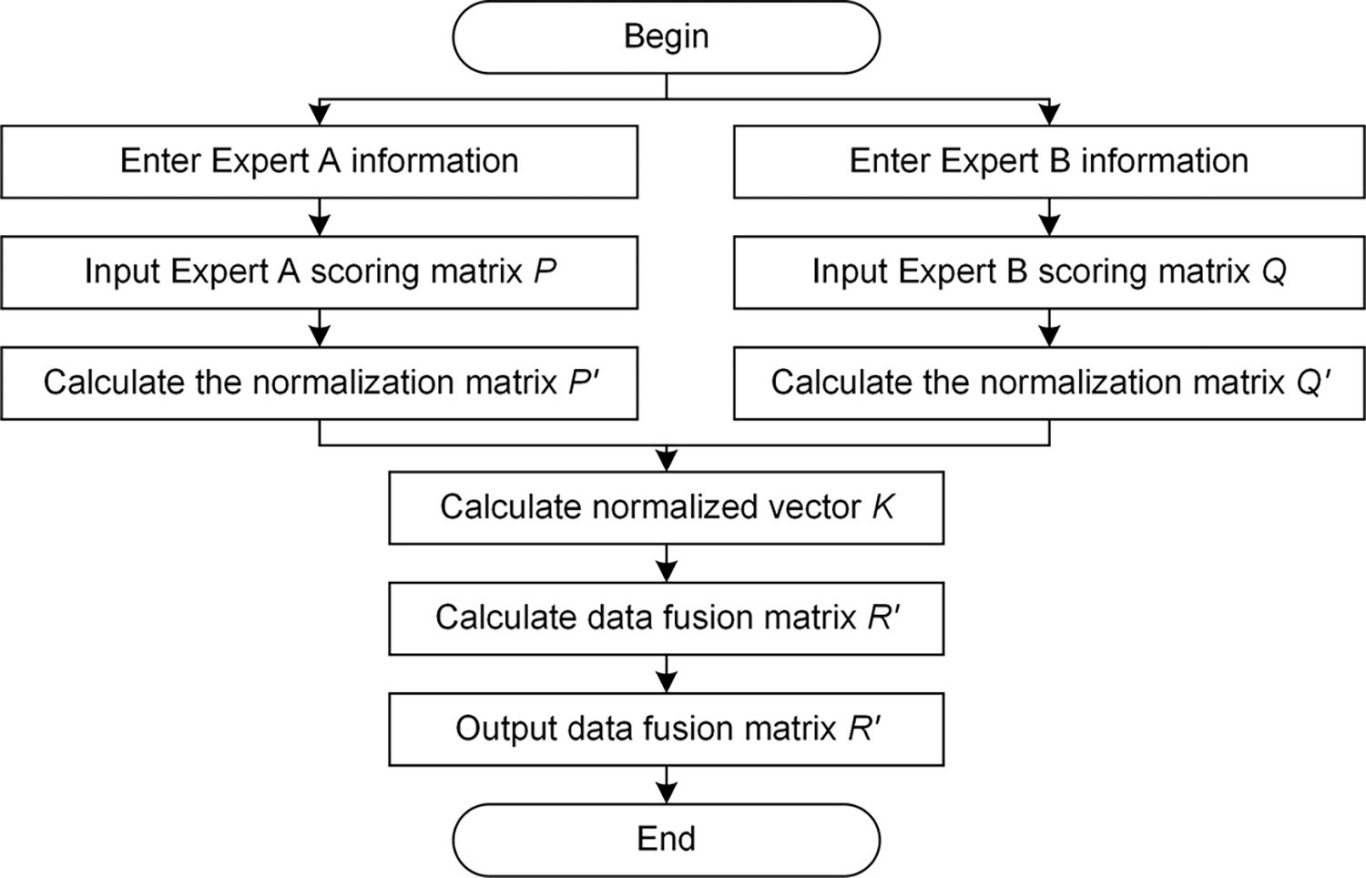
Process of data fusion.

**Fig 6:**
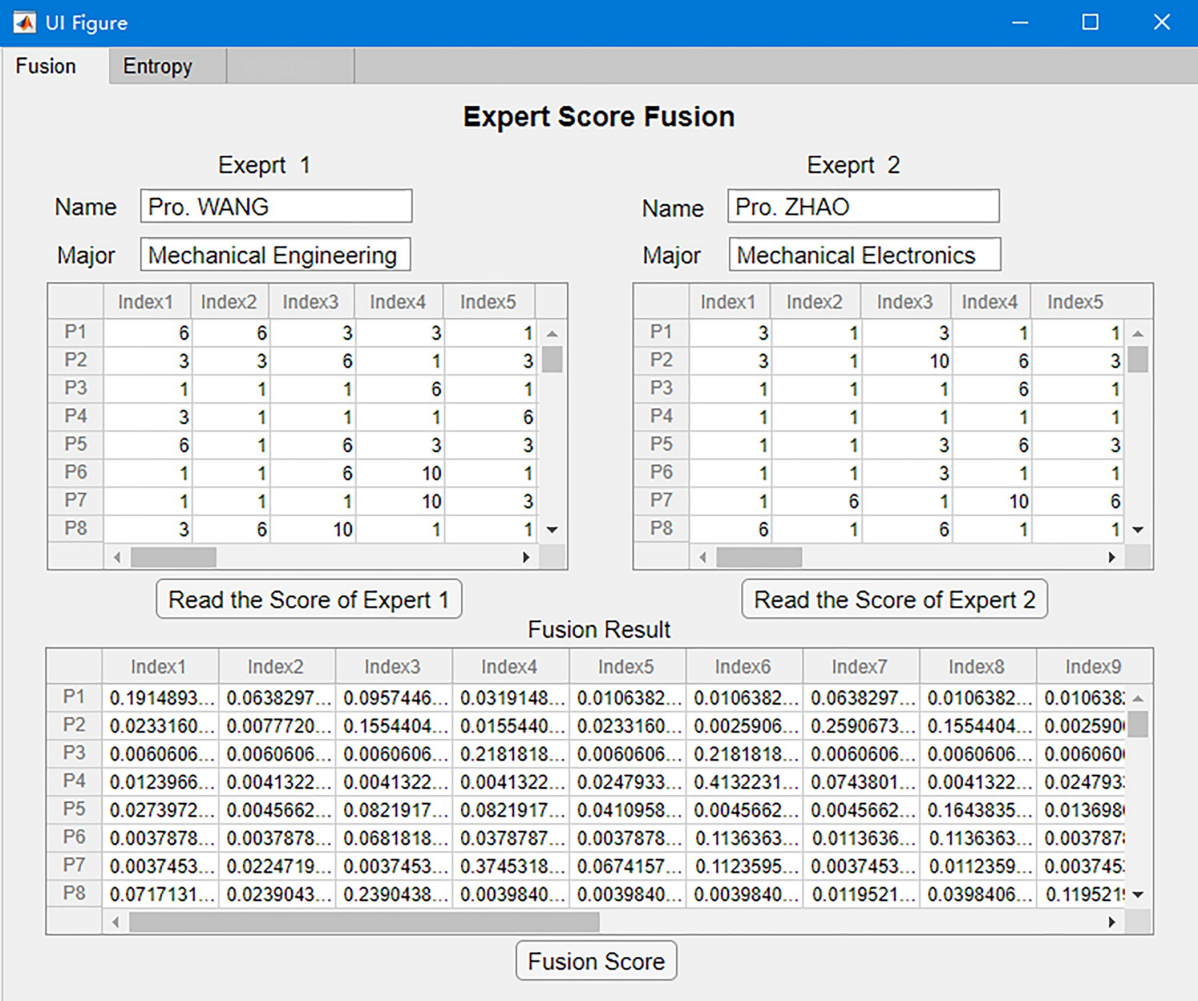
Two-dimensional D-S method operation interface.

In order to apply the method in the data fusion quickly and accurately, the process is coded in MATLAB. The process flow chart is shown in Fig 5. The data fusion of the two-dimensional matrix and operation interface is shown in Fig 6.

Because evaluation indicators have different influences on patents, different weights need to be assigned for indicators. Considering the large number of indicators and sufficient data after the fusion, the entropy method is used to decide weights of indicators [17].

Entropy method is a measure of the degree of system disorder, it can describe uncertainty of a system state. Therefore, the entropy value of each indicator is calculated by the entropy method. A smaller an entropy value indicates a more disorder degree of information, and greater weight of the indicator. The process of using the entropy method to calculate indicator weights are as follows.


$$s_{j}=\sum_{i=1}^{n}r_{ij}^{'}(i=1,2,3\ldots n)$$ (8)

$$r_{ij}^{''}=\frac{r_{ij}^{'}}{s_{j}}(i=1,2,3\ldots n;j=1,2,3\ldots m)$$ (9)

Matrix $R_{n\times m}^{''}$ is formed by using Formulas (8) and (9) to normalize columns of matrix $R_{n\times m}^{'}$. The logarithm of each element in matrix $R_{n\times m}^{''}$ is calculated, and matrix *L*_1×*m*_ is obtained by summation of columns as follows.


$$l_{j}=\sum_{i=1}^{n}\ln(r_{ij}^{''})\;\left(i=1,2,3\ldots n\right)$$ (10)

Weight *W*_*j*_ of each evaluation indicator is decided by Formulas (11) and (12). Weights of evaluation indicators are shown in Table 5. The partial data used for calculating indicator weights based on entropy method is shown in Fig 6. T_1_ to T_18_ is the notation for evaluating the weights of the indicators.


$$d_{j}=1-\frac{1}{\ln(n)}l_{j}$$ (11)

$$W_{j}=\frac{d_{j}}{\sum_{j=1}^{m}d_{j}}\;\left(j=1,2,3\ldots m\right)$$ (12)

**Table 5:** Weights of evaluation indicators (%).

Indicator	T_1_	T_2_	T_3_	T_4_	T_5_	T_6_
Weight	5.541	5.573	5.766	5.623	5.529	5.514
Indicator	T_7_	T_8_	T_9_	T_10_	T_11_	T_12_
Weight	5.571	5.432	5.462	5.529	5.525	5.542
Indicator	T_13_	T_14_	T_15_	T_16_	T_17_	T_18_
Weight	5.545	5.534	5.546	5.460	5.591	5.714

The technical value score of each patent can be obtained by multiplying the technical evaluation indicator weight *W*_*j*_ and the technical evaluation matrix $R_{n\times m}^{'}$. The calculation of technical value scores *H*_*n*×1_ is shown in Formula (13).


$$\begin{array}{l} H_{n\times 1}=R_{n\times m}^{'}\times W_{m\times 1}^{T}\\ \;=5.541\times T_{1}+5.573\times T_{2}+5.766\times T_{3}+5.623\times T_{4}+5.529\times T_{5}+5.514\times T_{6}\\ \;+5.571\times T_{7}+5.432\times T_{8}+5.462\times T_{9}+5.529\times T_{10}+5.525\times T_{11}+5.542\times T_{12}\\ \;+5.545\times T_{13}+5.534\times T_{14}+5.546\times T_{15}+5.460\times T_{16}+5.591\times T_{17}+5.714\times T_{18} \end{array}$$ (13)

**Fig 7:**
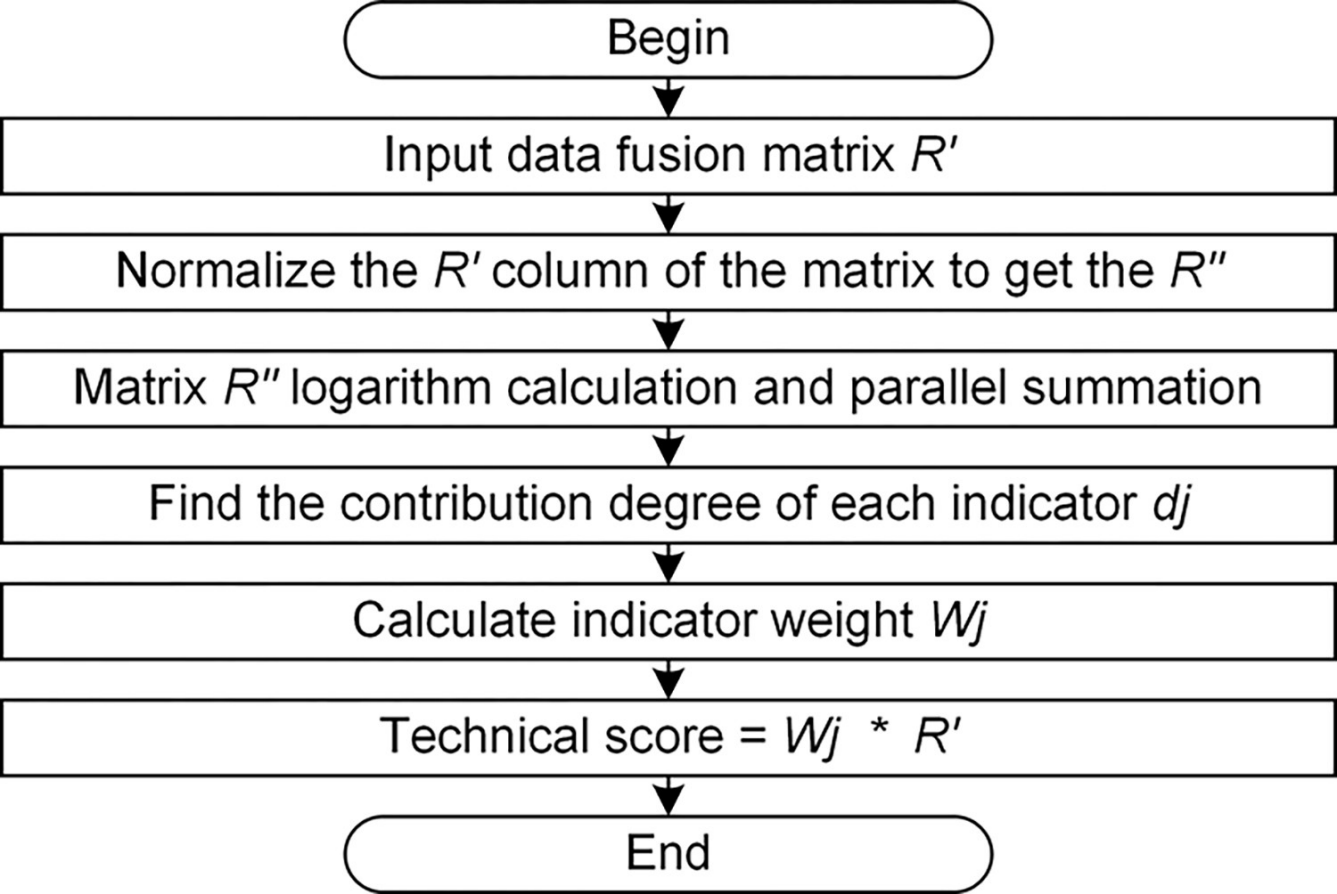
Process of technical value score calculation.

**Fig 8:**
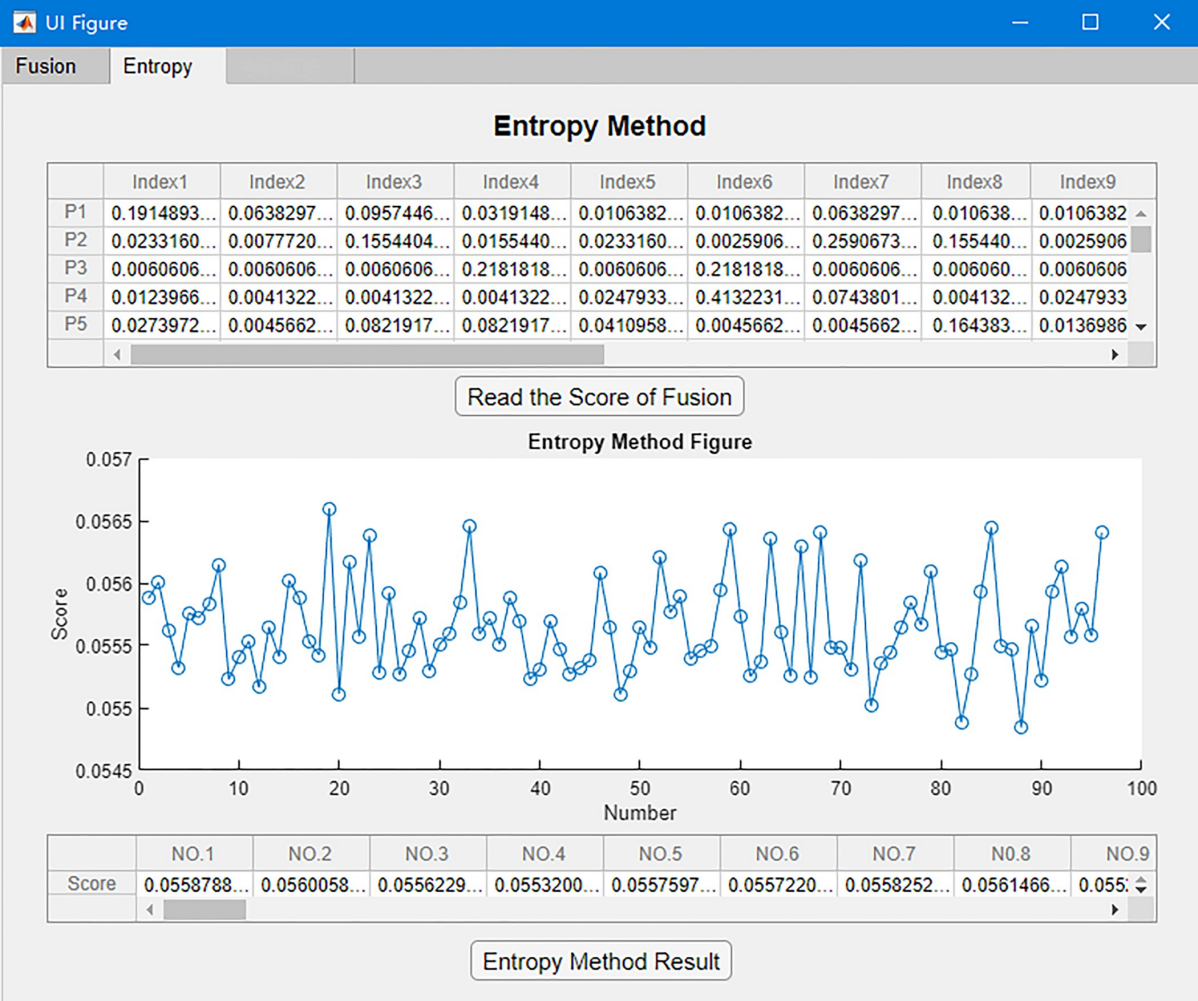
Operation interface for the entropy method to determine indicator weights.

A computer program is developed based on the above data process method using MATLAB to efficiently process a large number of patent data. It provides a unified and convenient tool to decide the indicator weight. The process of technical value score calculation is shown in Fig 7. The operation interface based on MATLAB is shown in Fig 8.

Matrix $R_{n\times m}^{'}$ is a combined scoring result from two experts. Each row vector in the matrix represents the technical value score of patent *P*_*i*_ under the evaluation indicator *T*_*j*_. The technical value score of each patent is multiplied by the weight of the corresponding indicator. The technical value score of each patent is then obtained by accumulating the multiplied results. Table 6 shows the technical value scores of some patent verification samples.

**Table 6:** Technical scores of patent verification samples (%).

Patent verification sample	H_1_	H_2_	H_3_	H_4_	H_5_	H_6_
Score	5.59	5.61	5.55	5.53	5.58	5.58
Patent verification sample	H_7_	H_8_	H_9_	H_10_	H_11_	H_12_
Score	5.57	5.62	5.52	5.53	5.55	5.52
Patent verification sample	H_13_	H_14_	H_15_	H_16_	H_17_	…
Score	5.56	5.54	5.61	5.60	5.55	…

## 4 Empirical analysis

The evaluation indicators and patent value evaluation methods constructed based on high-value patents in the field of mechanical can assist engineering designers in locating innovative technologies. In order to further verify the applicability of the proposed evaluation method to mechanical products, patents in the field of mechanical engineering are collected according to the IPC system as verification samples. Then, the patent values of the verification samples are calculated using the proposed method and the existing method to illustrate the effectiveness of the proposed patent technical value evaluation method.

**Fig 9:**
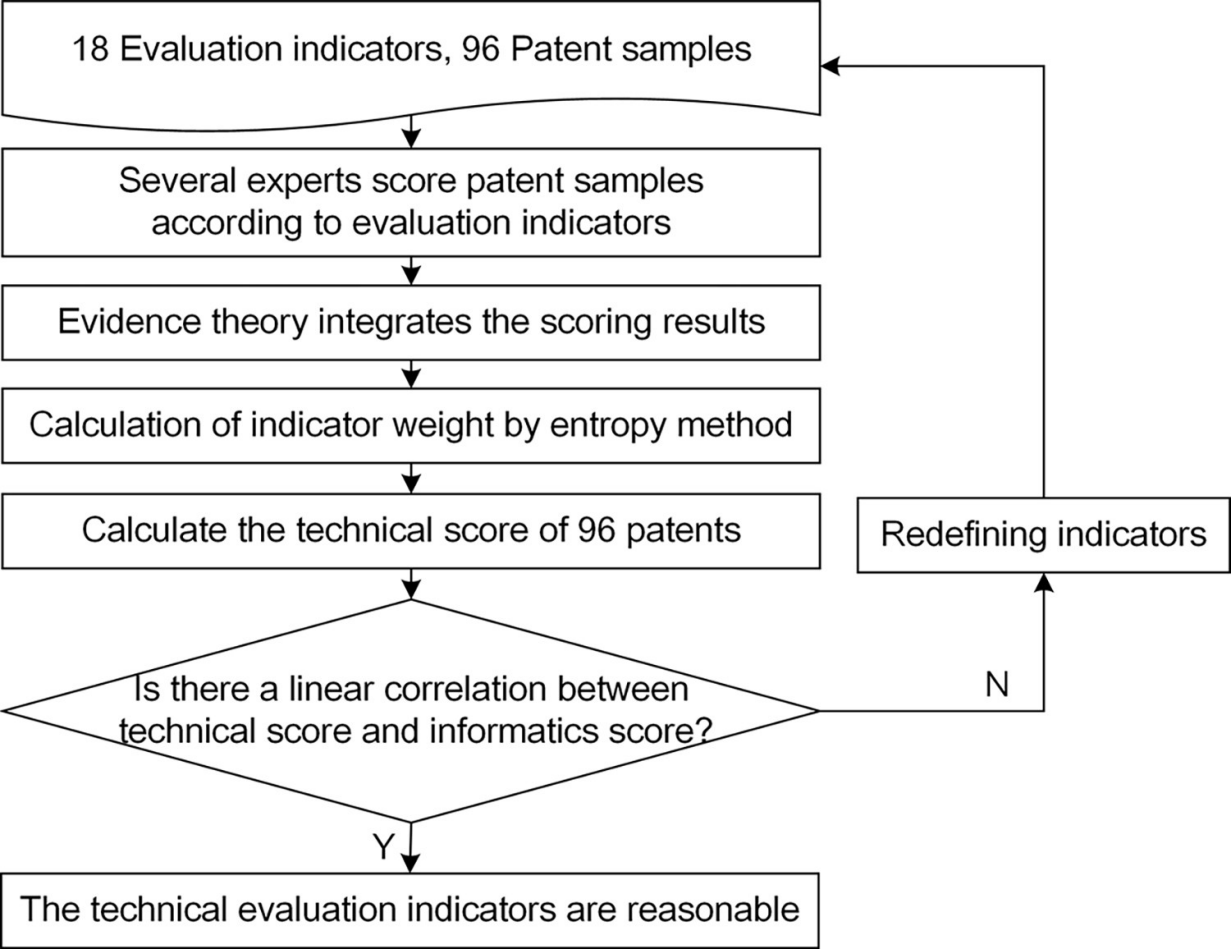
The process of empirical analysis on the proposed evaluation method.

The technical value score will be compared with the existing method to verify the rationality of the proposed evaluation method. The process of empirical analysis on the proposed evaluation method is shown in Fig 9.

Literatures [28,39,41] have demonstrated several information science evaluation methods of patent values. Huang [62] gives a more operationalized evaluation system that includes easy statistics indicator and detailed indicator weights. This section will introduce the patent value evaluation system given by Huang and use the system to calculate the patent scores of 96 verification samples. The calculated patent value scores will be compared with the proposed technical value scores to demonstrate the validity of the proposed evaluation method.

The patent value evaluation system given by Huang contains a total of three dimensions: economic, technical and legal, involving a total of 12 indicators. The patent value evaluation system and indicator weights are shown in Table 7.

**Table 7:** Patent value evaluation system and indicator weight.

First level	Secondary level	Third level	Indicator weight
Patent value	Technical value	Number of inventors C_1_	0.0810
		Citations C_2_	0.0751
		Number of classification numbers C_3_	0.0712
		Be cited C_4_	0.0797
	Economic value	Market application C_5_	0.0670
		Enterprise patents C_6_	0.0905
		Sales ratio C_7_	0.0879
	Legal value	Number of claims C_8_	0.0732
		Number of siblings C_9_	0.0879
		Manual pages C_10_	0.0922
		Survival period C_11_	0.0977
		License status C_12_	0.0964

Since the technical value of patents is focused on in this study. Therefore, only the four indicators belonging to the technical value in the above evaluation system are selected for calculation. Since the number of indicators used for calculation is changed, the weights of the four technical value indicators should be reallocated. After the recalculation of indicator weights, the formula of the existing patent value evaluation system is obtained, as shown in Formula 14.


$$y=0.263844\times C_{1}+0.244625\times C_{2}+0.231922\times C_{3}+0.259609\times C_{4}$$ (14)

The patent value of the verification samples is calculated by applying the patent value evaluation system. For the convenience of presentation, the above calculation results are referred to as the informatics scores. The partial informatics scores of the patent verification samples are shown in Table 8.

**Table 8:** Informatics scores of patent verification samples.

Patent verification sample	F_1_	F_2_	F_3_	F_4_	F_5_	F_6_	F_7_	F_8_
Scores	0.203	0.325	0.226	0.122	0.228	0.256	0.237	0.339
Patent verification sample	F_9_	F_10_	F_11_	F_12_	F_13_	F_14_	F_15_	…
Scores	0.233	0.239	0.245	0.248	0.241	0.223	0.336	…

Comprehensive technical scores and informatics scores evaluate the patent value from two different dimensions. Only when there is a linear correlation between the scores of the two evaluation methods, can we prove the consistency of the evaluation results of the two methods, and the rationality of the proposed indicators and their weights.

In order to compare the correlation between the two sets of data visually, patent values calculated by Formulas (13) and (14) are plotted in a same coordinate system. Because data magnitudes of scores from the two methods are different, it is difficult to map them in the same coordinate system. Therefore, technical score *H*_*i*_ and informatics score *F*_*i*_ are normalized respectively. Through the data normalization, it can ensure that the two sets of data can be compared conveniently without losing their own information. The data are normalized using Formula (15).


$$h_{i}^{'}=\frac{h_{i}-\min(h_{i})}{\max(h_{i})-\min(h_{i})}\;\left(i=1,2,3\ldots n\right)$$ (15)

**Fig 10:**
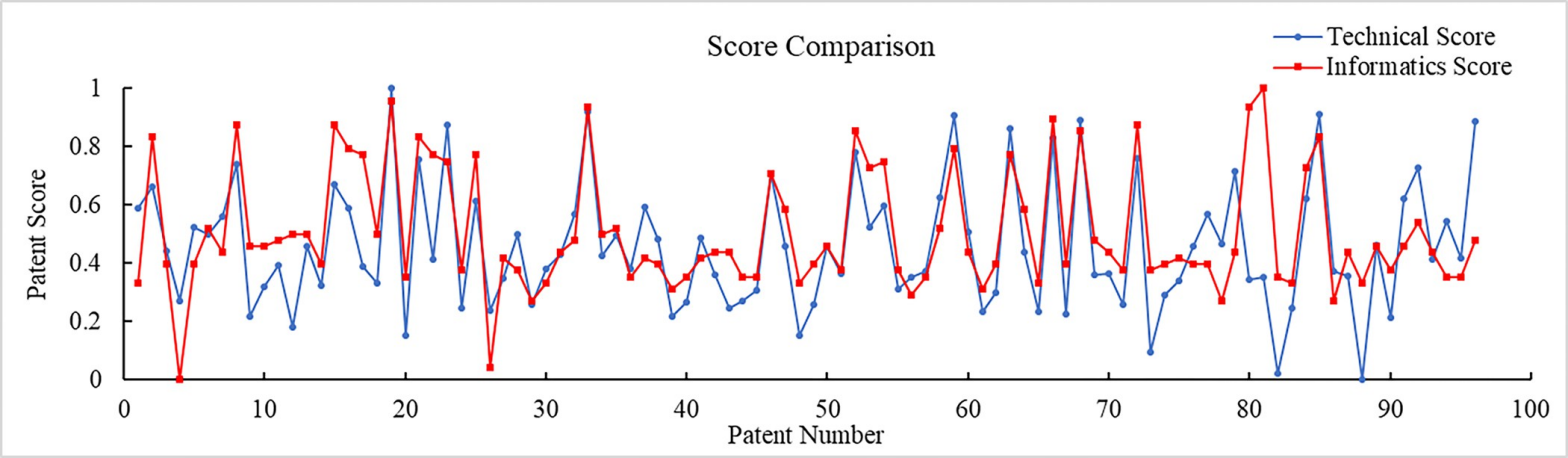
Comparison of technical scores and informatics scores.

Where 0 ≤ *h*_*i*_*’* ≤1. After the normalization of two sets of data, the dimension of each set is eliminated and the order of magnitude of the two sets is unified. The results are shown in Fig 10. It can be seen that the trend of the two sets of data is basically the same. The highest and lowest scores in the same class appear in the same position, and results of the two evaluation methods have a positive correlation.

In order to show the correlation between the two data in further detail, the Pearson correlation coefficient method is introduced. It can be measure the correlation between two sets of data *H* and *F*, as shown in Formula (16) for Pearson correlation coefficient.

$$r_{H,F}=\frac{\sum_{i=1}^{n}\left(H_{i}-\bar{H})(F_{i}-\bar{F}\right)}{\sqrt{\sum_{i=1}^{n}{\left(H_{i}-\bar{H}\right)}^{2}}\sqrt{\sum_{i=1}^{n}{\left(F_{i}-\bar{F}\right)}^{2}}}$$ (16)

where *r*_*H*,*F*_ is the Pearson correlation coefficient between data *H* and *F*, $\bar{H}$ is the mean of data *H*, and $\bar{F}$ is mean of data *F*.

Pearson correlation coefficient is usually expressed as *r* in the range of [–1, 1]. A positive *r* means that the two variables are positively correlated, and vice versa. A correlation coefficient *r* with a value closed to 1 (-1 or +1) indicates a strong correlation of two data sets, and a week correlation when the value is close to 0. The two variables are completely linear when *r* = 1 or -1. The value of *r* can be used to describe strength of the correlation as shown in Table 9.

**Table 9:** Pearson correlation coefficient range and meaning.

Range	Degree of correlation
|*r*|<0.3	Weak correlation
0.3≤|*r*|<0.5	Low correlation
0.5≤|*r*|<0.8	Significant correlation
0.8≤|*r*|<1	High correlation

SPSS statistical software [63] is used to calculate the correlation between the two sets of data in Tables 6 and 7. As the technical themes of seventeen classes are quite different, the Pearson correlation coefficient is calculated for patents in each class. Table 10 shows the calculation results.

**Table 10:** Pearson correlation coefficients.

Class	Correlation coefficient	Degree of correlation	Class	Correlation coefficient	Degree of correlation
1–7	0.8505	High correlation	57–66	0.9332	High correlation
8–17	0.8347	High correlation	67–75	0.9620	High correlation
18–22	0.9223	High correlation	76–79	0.6754	Significant correlation
23–26	0.8589	High correlation	81–82	0.6754	Significant correlation
28–43	0.8332	High correlation	83–86	0.9281	High correlation
44–47	0.9679	High correlation	87–93	0.9615	High correlation
48–53	0.9209	High correlation	94–96	0.9657	High correlation
54–56	0.9544	High correlation			

Based on Pearson correlation coefficients, it can be seen that the correlation coefficients are all greater than zero, which indicates that there is a positive correlation between the technical score and informatics scores. 86.67% of the classes have a high correlation. There is no low correlation and weak correlation. Therefore, it can be seen that the proposed method can reflect the patent technical value. It proves that the proposed method is highly correlated and consistent with the existing method, which verifies the rationality of the proposed technical evaluation method.

## 5 Discussion

The proposed patent technical value evaluation method, which contains 18 indicators, can assist engineering designers in product development activities. The effectiveness of the proposed patent value evaluation method is verified through empirical analysis. The proposed method provides a new perspective for the evaluation of patent technical value from the perspective of technical characteristics of high-value patents. In order to enhance the convenience of the proposed evaluation method in application, a patent value evaluation model with a smaller number of indicators will be explored in this section. The method of cultivating high tech-value patents will also be described in this section, which will in turn illustrate the process of applying the proposed tech-value evaluation method in the engineering field.

### 5.1 Five dimensional evaluation formula

Based on above solutions of using the entropy method, evidence theory and correlation analysis, proposed evaluation method with 18 indicators can describe patent technical values. Considering the detailed definition and large number of indicators, it is necessary to compare evaluation rules carefully when assessing the patent value. In order to reduce the workload of users and enhance the operability of the evaluation method. The 18 indicators are dimensionally reduced by using principal component analysis and factor analysis method based on patent scoring data.

**Fig 11:**
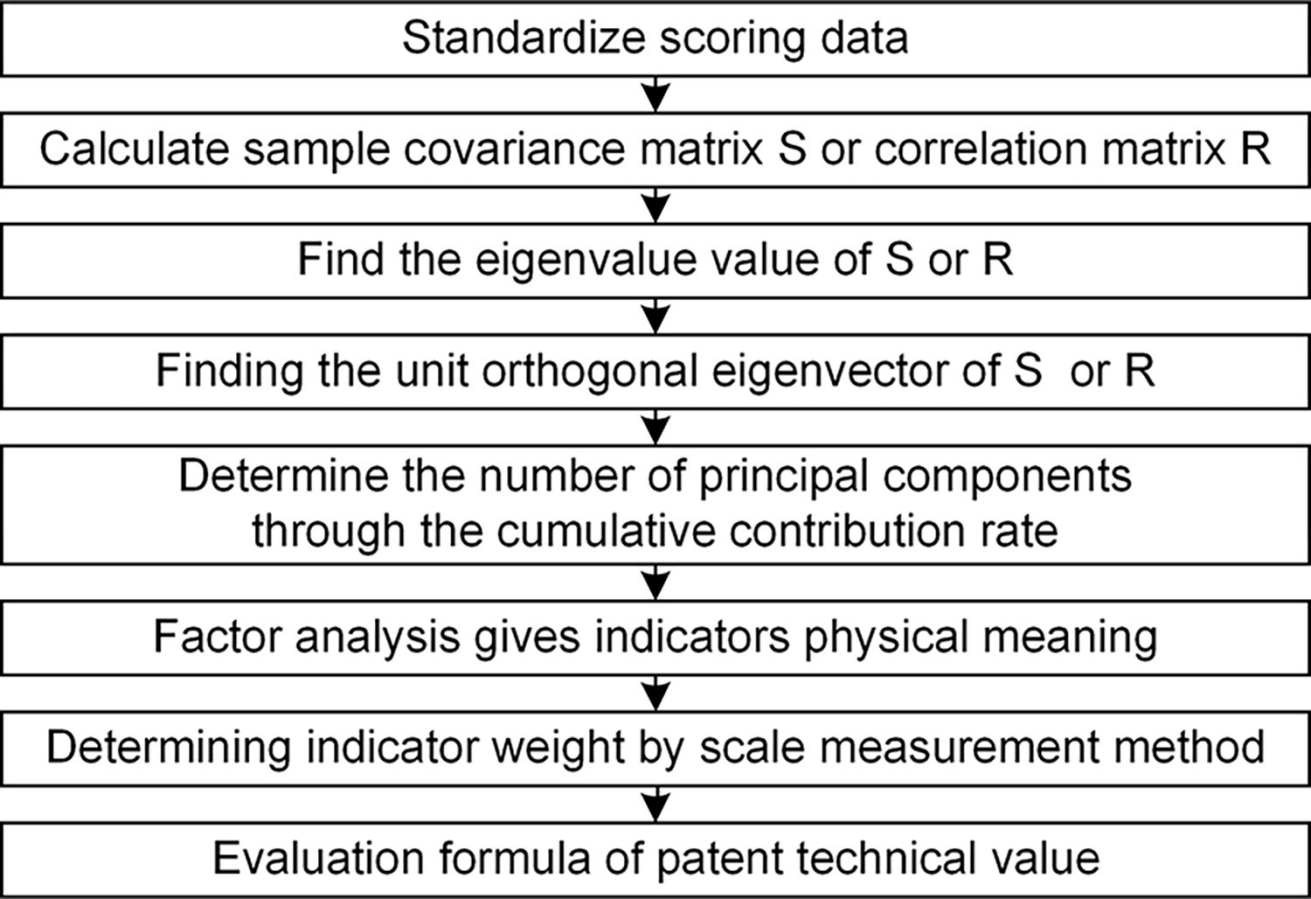
Process of patent technical value evaluation.

The idea of the principal component analysis is to recombine original indicators into a set of independent indicators, and retain as much information as possible. Factor analysis is then applied to give actual physical meaning to the reduced dimensional indicators, enhancing the designer’s operability of the evaluation indicators. Principal component analysis and factor analysis are used to propose a patent technical value evaluation formula. The specific process is shown in Fig 11.

Table 11 shows the results of processing patent scoring data by the principal component analysis in SPSS software [64]. Data in the table show that the eigenvalue value of the first five principal components is greater than 1, and their cumulative contribution rate reaches 70.694%, which meets the requirement that the cumulative contribution rate in statistics is greater than 70%, so the number of principal components is selected as 5 [65]. These principal components represent the information of 18 original variables.

**Table 11:** Results of principal component analysis.

Component	Initial eigenvalue			Cumulative contribution rate		
	Total	Variance %	Accumulate %	Total	Variance %	Accumulate %
1	3.842	21.346	21.346	2.808	15.598	15.598
2	3.295	18.307	39.654	2.759	15.328	30.926
3	2.554	14.189	53.843	2.677	14.874	45.800
4	1.854	10.301	64.144	2.252	12.510	58.310
5	1.179	6.550	70.694	2.229	12.385	70.694
6	0.859	4.770	75.464			
7	0.757	4.204	79.667			
8	0.631	3.508	83.175			
9	0.515	2.859	86.034			
10	0.509	2.827	88.861			
11	0.461	2.559	91.420			
12	0.387	2.150	93.570			
13	0.318	1.766	95.336			
14	0.238	1.321	96.657			
15	0.225	1.251	97.908			
16	0.200	1.112	99.021			
17	0.096	0.532	99.552			
18	0.081	0.448	100.00			

Because the 5 principal components selected are the linear combination of the 18 original variables, these principal components no longer have engineering practical significance. Considering that parameters without practical significance are difficult to be used by engineers, engineering interpretations should be given to the 5 principal components. Factor analysis is an effective tool to describe the actual meaning of each principal component. A statistical test of the correlation coefficient matrix should be performed before doing factor analysis. If the correlation between the factors is high, then there is a shared common factor between the variables and it is suitable for factor analysis. The results of the Bartlett’s test and Kaiser-Meyer-Olkin test based on the determinant of the correlation coefficient matrix are shown in Table 12.

**Table 12:** KMO and Bartlett tests.

Kaiser-Meyer-Olkin test		0.766
	Approximate chi square	556.523
Bartlett test	Degree of freedom	15
	Significance	0.00

The KMO statistic is an indicator used to compare the simple correlation coefficient and partial correlation coefficient between the variables, and it takes values between 0 and 1. A KMO statistic value greater than 0.5 makes the data suitable for factor analysis. The result of Bartlett’s test is less than 0.05, then there is correlation between the original variables and the data is suitable for factor analysis. Therefore, Kaiser orthogonal rotation method in the factor analysis is used to process the data. The ultimate goal is to highlight the relationship between each common factor and variables with the large load. The interpretation of each common factor can be explained reasonably by these variables. The matrix converges after six times of the orthogonal rotation, and the results are shown in Table 13.

**Table 13:** Factor analysis results.

Technical indicator number	Five common factors				
	1	2	3	4	5
A	0.015	-0.095	-0.206	-0.103	0.695
B	-0.241	0.757	-0.341	-0.192	-0.113
C	-0.313	0.073	0.006	0.032	0.811
D	-0.461	-0.207	0.606	-0.098	-0.167
E	0.775	-0.135	0.060	-0.096	-0.150
F	-0.026	-0.101	0.821	0.081	-0.077
G	0.733	0.068	0.085	0.011	-0.046
H	-0.128	-0.175	-0.247	0.740	0.036
I	0.170	0.855	0.040	-0.178	0.033
J	-0.025	-0.154	0.082	0.857	-0.006
K	0.124	-0.059	0.821	-0.048	-0.134
L	0.760	0.032	-0.255	-0.110	-0.106
M	-0.241	-0.189	0.730	-0.189	0.003
N	0.704	-0.009	-0.187	-0.177	-0.312
O	0.124	0.847	-0.066	0.024	0.086
P	-0.116	-0.060	-0.018	0.862	-0.080
Q	-0.202	0.740	-0.283	-0.199	-0.114
R	-0.195	0.003	-0.070	0.017	0.928

From results of the factor analysis, indicators with a large load on factor 1 are E, G, L, and N. Indicators with a large load on factor 2 are B, I, O, and Q. Indicators with a large load on factor 3 are D, F, K, and M. Indicators with a large load on factor 4 are H, J, and P. Indicators with a large load on factor 5 are A, C, and R.

According to distributions of load indicators on each principal component. The practical significance to each principal component can be obtained as shown in Table 14.

**Table 14:** Common factor and its specific meaning.

NO.	Common factor	Description
1	Deformation assembly factor	Realizing deformable design to adapt to various working conditions under the premise of ensuring assembly accuracy
2	Human-computer interaction factor	In line with the purpose of man-machine engineering, convenient for user operation and use
3	Structural improvement factor	Improve mechanical structure and improve stability and reliability
4	Process improvement factor	Optimize process route and improve process procedure
5	Functional principle factor	By changing the physical principle of realizing function, a new design scheme is obtained

Based on the principal component analysis and factor analysis, five evaluation indicators are obtained as follows: deformation assembly, human-computer interaction, structure improvement, process improvement and functional principle. When using evaluation indicators to assess the patent value, different indicators have different influence on the patent value. Different weights of each indicator should be assigned. Common factors are weighted by the scale measurement method of a pairwise comparison [66], as shown in Table 15 for the weight of each common factor.

**Table 15:** Common factor weight from scale measurement.

Common factor	*m*_1_	*m*_2_	*m*_3_	*m*_4_	*m*_5_	Total score	Proportion
*m*_1_	0	0	0	0	1	1	1/9
*m*_2_	0	0	0	1	0	1	1/9
*m*_3_	0	0	0	1	1	2	2/9
*m*_4_	0	1	1	0	0	2	2/9
*m*_5_	0	1	1	1	0	3	3/9

Patent values are obtained by multiplying the evaluation indicator and its weight. Formula (17) shows the five dimensional evaluation formula of patent technical value.

$$M_{i}=0.11\times m_{i1}+0.11\times m_{i2}+0.22\times m_{i3}+0.22\times m_{i4}+0.33\times m_{i5}$$ (17)

where, *M*_*i*_ is the patent value in technology (*i* = 1,2,3…*n*), *m*_*i*1_ is the deformation assembly factor, *m*_*i*2_ is the human-computer interaction factor, *m*_*i*3_ is the structural improvement factor, *m*_*i*4_ is the process improvement factor, and *m*_*i*5_ is the functional principle factor.

### 5.2 Empirical analysis for the five dimensional

In the previous section, 18 evaluation indicators are reduced to 5 common factors by the principal component analysis and factor analysis method. Although the cumulative contribution rate of the principal component analysis has met the requirement of more than 70% of the cumulative contribution, there is still a small amount of information loss. Therefore, it is necessary to do further empirical analysis for the effectiveness of the patent technical value evaluation Formula (17).

Patent samples of No. 1–7 as an example are verified, invite experts score the patent verification samples by referring to scoring rules of the technology genealogy tree and fuse the scoring results. The patent scores are finally calculated using Formula (17), and the rationality is verified by the correlation evaluation with the result of Formula (13). Patent technical scores based on the five dimensional evaluation indicators are shown in Table 16.

**Table 16:** Patent scores after indicator dimension reduction.

Patent samples	Deformation assembly	Human-computer interaction	Structural improvement	Process improvement	Functional principle	Patent score
CN109404046A	0.343	0.057	0.343	0.171	0.086	0.187
CN109162762A	0.380	0.034	0.380	0.068	0.137	0.191
CN109356674A	0.746	0.007	0.067	0.045	0.134	0.153
CN109268087A	0.305	0.305	0.305	0.076	0.008	0.155
CN109356682A	0.040	0.441	0.040	0.441	0.040	0.173
CN109339902A	0.578	0.006	0.208	0.104	0.104	0.169
CN109372617A	0.271	0.271	0.226	0.226	0.008	0.163

**Fig 12:**
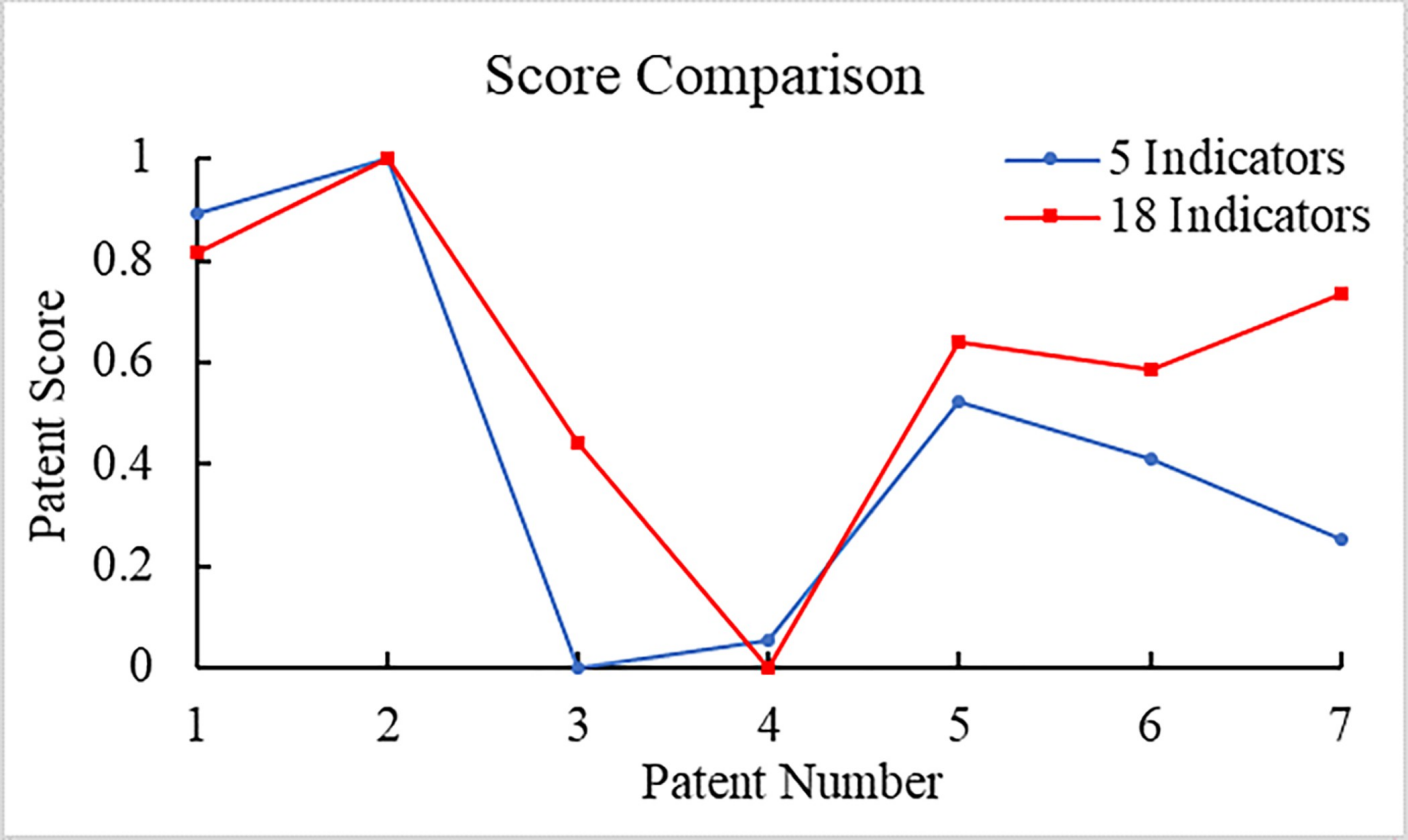
Score comparison.

To compare the correlation between two sets of data, the patent scores in Tables 6 and 8 are displayed graphically. First of all, the two evaluation scores are normalized to eliminate dimensions for unifying the magnitude. Processed data are then drawn in the same coordinate system as shown in Fig 12.

In order to calculate the correlation between two sets of scores accurately, the correlation analysis of the scores before and after the indicator dimensionality reduction is conducted by using Formula (16) and SPSS statistical analysis software. Results of the correlation analysis are shown in Table 17.

**Table 17:** Correlation analysis.

Indicator	Correlation analysis	Eighteen technical indicator	Five technical indicator
Eighteen technical indicator	Pearson correlation	1	0.808^*^
	Significance (Bilateral)	0	0.028
	Number	7	7
Five technical indicator	Pearson correlation	0.808*	1
	Significance (Bilateral)	0.028	0
	Number	7	7

It can be seen from the analysis results that the trend of the two sets of data is similar basically, and the Pearson correlation coefficient of the data before and after the dimensionality reduction is 0.808, namely |*r*| = 0.808. It indicates that scores of the two evaluation methods are highly positively correlated. The P value of the bilateral test is 0.028 < 0.5, which means rejecting the original hypothesis that there is no correlation between the two sets of date, which strengthens the consistency of results before and after the indicator dimension reduction. The correlation coefficient of a single asterisk mark in the table indicates that the significance level is below 0.05. The correlation coefficient is significant with a high correlation. In conclusion, it can be seen from the analysis that there is a significant positive correlation between the evaluation formula before and after the indicator dimension reduction. The calculation results of the two formulas are highly consistent, which proves the rationality of the five dimensional evaluation method.

### 5.3 Application process of the proposed method

Both the 18-indicator and the 5-indicator patent technical value evaluation methods can provide R&D directions for engineers’ product design activities. In this section, cutting machine will be used as an example to show the application of the proposed evaluation method in the improvement of product structure. The proposed method can assist designers to enhance the innovativeness of the designed products and has strong application value in engineering practice.

Cutting machine is also called abrasive-disk cutter. The abrasive-disk cutter is used in construction, petrochemical industry, mechanical metallurgy and other fields. It is a basic and important processing tool of machine processing. It has advantages such as the simple structure, convenient assembly, easy to carry, and low price. However, the existing equipment has some shortcomings in unstable loading, high noisy and burn of pipe wall due to friction. Therefore, the proposed five dimensional evaluation method will be applied to design a more innovative abrasive-disk cutter.

Patent retrieval is conducted using the keyword of abrasive-disk cutter, 403 authorized invention patents are found. The technical value of each patent is evaluated through Eq (16). The patent with the highest score is identified as CN105798390B. The patent uses the principle of water cutting to cut the steel pipe. The high-speed jet water can remove the burr on the inner wall of the steel pipe, and the low-temperature water takes away the heat generated in the cutting process, ensuring the quality of the cut steel pipe.

**Fig 13:**
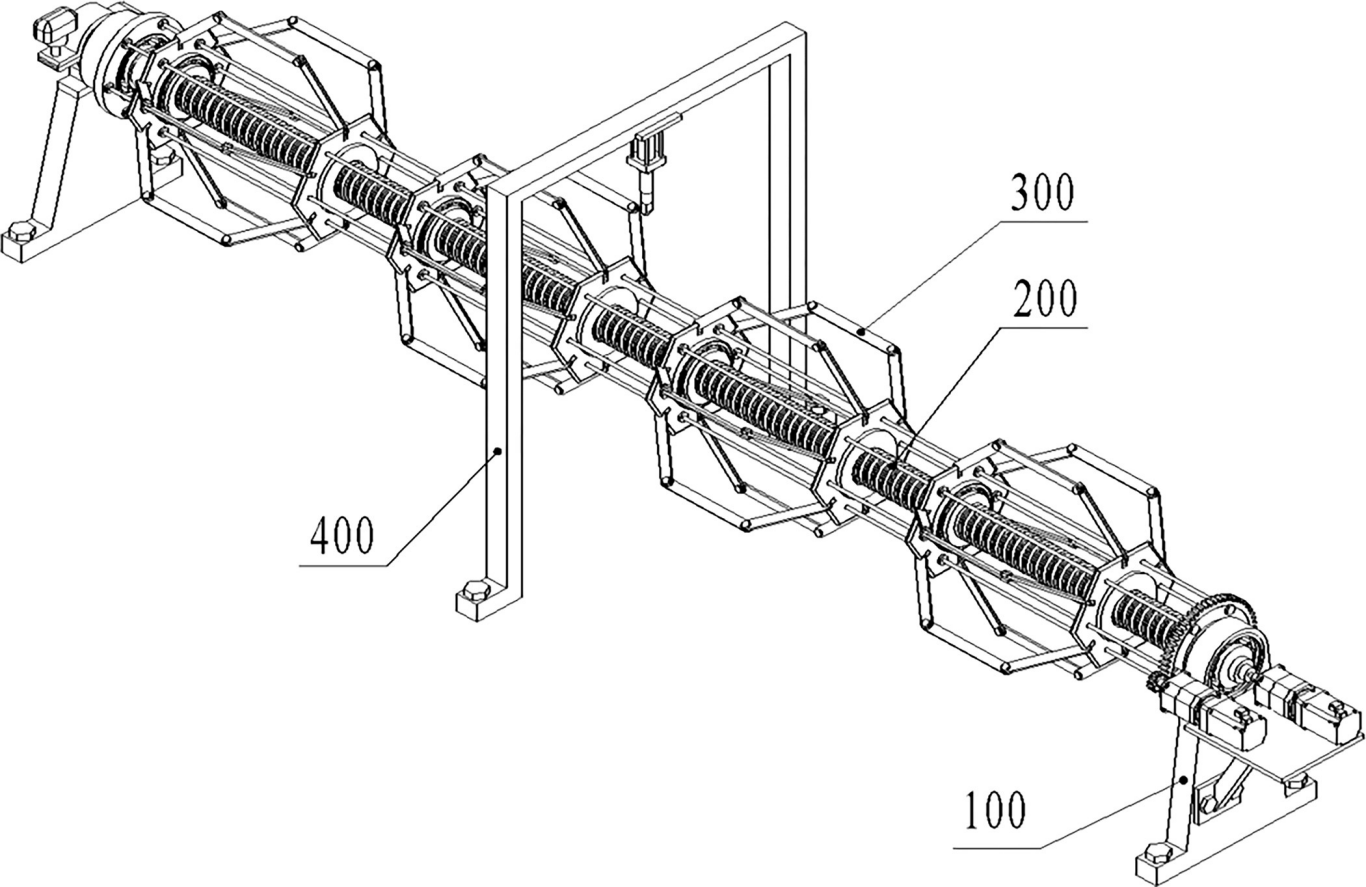
New product design structure. (100- Support device, 200- Lead screw device, 300- Expansion device, 400- Cutting device).

However, this patented cutter can only cut steel pipes with a fixed diameter, which has strict restrictions on the size and installation location of pipe fittings, so the score under deformation assembly indicator is only 3 points. Based on the analysis of disadvantages of the patent, it is determined that the first problem need to be solved is "adapting to multi-path cutting". Therefore, a lead screw type clamping structure is proposed, which has the function of automatically centering the cut steel pipe to reduce the installation and positioning requirements. The new designed structure can cut the steel pipe with diameters of 60-120mm using the hydraulic feed cutter, which solves the identified problem. Improvements that take evaluated patent as the design starting point involve higher novelty in structure. The scheme structure of the improved design is shown in Fig 13.

## 6 Conclusions

High value patent is an important embodiment of product innovation, a symbol of the birth of innovative technology, and a source to stimulate innovation. An effective evaluation method is proposed in this paper to identify patent values from the technical point of view. First, Construct a patent technical value evaluation model. A method of mining high technology value patents based on literature search is proposed. The technical features embedded in the high technology value patents are identified and mapped into indicators used to evaluate the technical value. The weights of each indicator are calculated based on the entropy method, and a patent technical value evaluation model is constructed. Second, Empirical analysis of the patent technical value evaluation model. A method is proposed to extract patents in the field of mechanical as a verification sample. An evidence theory method of fusing two-dimensional matrix data is proposed to fuse the scores of two experts on the technical value of patents. The proposed method is compared with existing methods to demonstrate the effectiveness of the proposed method. Third, Application method of the proposed model. Based on the principal component analysis and factor analysis, an evaluation model containing five indicators is proposed to make the proposed method easier to use by engineers. The application process of the proposed method in product design is demonstrated with the cutting machine as an example.

The contributions of the proposed method mainly include the following aspects. (1) The evaluation method constructed based on high technology value patents can assist the enterprise designers to determine the direction of product development and carry out product innovation design. The proposed technical value evaluation indicators that are not limited to statistical significance can be directly used in the product design process. (2) The evaluation method containing 5 indicators can quickly screen out high-value patents, and then use the evaluation method containing 18 indicators to calculate the patent value in detail. It achieves to ensure the accuracy of the calculation results and at the same time improve the computing efficiency. (3) An evidence theory calculation method oriented to two-dimensional data is proposed to realize the fusion of scoring results of two experts and reduce the subjectivity of scoring. The constructed automation program improves the efficiency of processing patent data.

Future research will expand the applicability of the proposed method. Based on the ideas of the proposed research method, it is expected to propose a set of evaluation paths for patent technical value in other disciplines such as chemical engineering or electrical engineering. In the future research, more engineering cases need to be applied for the feedback improvement of the proposed method. Although a two-dimensional data fusion method is proposed, enhancing the objectivity of scoring needs to be further investigated in the future.
